# Author Index

**DOI:** 10.1038/sj.bjc.6601995

**Published:** 2004-06-22


					Author Index
Abdel-Fattah R S11 (2.7)
Aboagye E S45 (P76)
Aboagye EO S44 (P75)
Abson C S80 (P176:2)
Addison K S7 (S21)
Afseth J S22 (8.4)
Agrawal R S80 (P176:2)
Ahern R S21 (7.6), S43 (P72:1)
Aherne W S7 (S22)
Ahmad S S15 (4.5), S15 (4.6)
Ahmad T S8 (1.3), S42 (P70)
Ah-See MW S16 (5.4), S22 (8.1)
Airley R S48 (P81), S49 (P82),
S68 (P146)
Airley RE S52 (P91)
Aitchison M S32 (P33), S32
(P34)
Aladin F S66 (P136)
Albrecht M S58 (P108)
Alexander FE S43 (P72:3)
Ali S S71 (P157:1)
Alison N S27 (P15)
Allen G S8 (1.4)
Allibone R S44 (P74)
Al-Mufti R S24 (P3)
Al-Nahhas A S44 (P75)
Amlot P S59 (P113)
An Q S71 (P155)
Andersen JS S73 (P157:7)
Anderson E S9 (1.8)
Anilkumer N S72 (P157:3)
Anson K S36 (P48)
Anstruther PT S58 (P109)
Anthony R S56 (P107)
Anyamene NA S35 (P46)
Anyamene NA S38 (P57)
Appleyard MVCL S62 (P128)
Argraves WS S70 (P152)
Armstrong K S67 (P142)
Ashcroft M S7 (S22)
Ashley S S15 (4.8)
Ashley S S27 (P16)
Ashton A S19 (6.8)
Ashworth A S5 (S11)
Atherton J S12 (3.2)
Atkin KM S39 (P59:1)
Attard G S25 (P5)
Awan A S11 (2.6)
Azadeh B S31 (P29)
Azribi F S23 (8.7)
Bacon AL S10 (2.3)
Baig N S18 (6.1), S47 (P77)
Bailey T S34 (P42)
Bailey TA S54 (P98)
Balkwill F S74 (P157:12)
Balkwill FR S57 (P107:5), S74
(P157:11)
Balroop S S48 (P80:3)
Banavali S S55 (P104)
Banks RE S33 (P36)
Banwell CM S22 (8.3)
Baokbah TAS S14 (4.1)
Barber P S60 (P117)
Bardwell H S73 (P157:6)
Barnes NLP S9 (1.8)
Barnwell JM S77 (P168)
Barr H S55 (P105)
Barrett-Lee P S80 (P176:2)
Bartelemes L S40 (P61)
Bartsch D S31 (P29), S32 (P31)
Bartsch DK S40 (P64)
Barvaux VA S61 (P123)
Batley S S57 (P107:6)
Batt LA S79 (P173), S79 (P174)
Baughan C S30 (P26)
Bayne M S8 (1.1)
Beckett P S8 (1.2)
Begent RH S59 (P113)
Begent RHJ S8 (1.2), S63 (P130)
Begg AC S6 (S14)
Bell Z S44 (P72:5)
Benepal T S25 (P5)
Bennett E S66 (P136)
Ben-Shlomo Y S35 (P45), S36
(P48)
Bentzen S S13 (3.6), S S19 (6.8),
S80 (P176:3)
Bentzen SM S16 (5.4)
Berdini V S56 (P107)
Beresford M S29 (P22)
Berrieman HK S76 (P164), S76
(P165)
Bertram E-L S29 (P23)
Bethel JA S39 (P60)
Bhatia KG S55 (P104)
Bhattacharyya M S24 (P3)
Bibby M S64 (P130:3)
Bibby MC S49 (P84), S58 (P110)
Bidmead M S79 (P176)
Biglari A S56 (P107:1)
Birch JM S43 (P72:3)
Bishop AJ S43 (P72:2)
Bissett D S8 (1.4), S12 (3.4)
Bissett JD S14 (4.3), S14 (4.4)
Blackall JM S15 (4.6)
Blackhall FH S14 (4.2), S29
(P22:1), S66 (P137)
Blair GE S65 (P132), S65 (P134)
Blake N S66 (P136)
Blake P S79 (P176)
Blakey DC S46 (P76:5)
Bliss J S19 (6.8), S80 (P176:2),
S80 (P176:3)
Bliss JM S1 (CT3)
Bliss P S80 (P176:2)
Bloomfield D S80 (P176:2)
Blow JJ S73 (P157:7)
Bogdanova TI S39 (P60), S42
(P72)
Bonde P S44 (P72:5)
Bottley G S65 (P132)
Boulos PB S38 (P56)
Boult J S66 (P138)
Bower M S13 (3.7)
Bowman A S22 (8.4)
Bown SG S35 (P43), S35 (P44)
Boxer G S8 (1.2)
Boyd M S18 (6.1), S19 (6.5), S19
(6.6), S47 (P77), S47 (P78),
S48 (P80:1)
Boyd MT S65 (P131)
Boylan K S7 (S21)
Boyne JR S65 (P133), S66 (P135)
Bradbury P S28 (P18), S28 (P19)
Bradley RG S72 (P157:2)
Bradshaw TD S9 (1.5)
Braidwood L S53 (P97)
Branston LK S79 (P174)
Brant DP S77 (P168)
Bray SE S62 (P128)
Bridges EM S58 (P110)
Bridle H S28 (P17)
Brimmell M S63 (P130:2)
Brittain-Dissont SJ S52 (P91)
Broad B S19 (6.8)
Brock SJ S30 (P26)
Bromwich EJ S32 (P33), S32
(P34)
Brooks M S34 (P40)
Broomhead DM S54 (P98)
Brown JE S20 (7.3), S61 (P122)
Brown L S50 (P87), S50 (P89)
Brown MD S62 (P127)
Brown R S23 (8.5)
Brown R S53 (P97), S61 (P123)
Brown S S64 (P130:6)
Brown SJ S59 (P114), S59 (P115)
Brunt AM S1 (CT3)
Brunt M S80 (P176:2)
Bubnovskaya LN S49 (P83)
Buckley JR S25 (P6)
Bullard SH S30 (P27)
Bundred NJ S9 (1.8), S27 (P13)
Burchill SA S17 (5.6), S58
(P110), S65 (P133), S66
(P135), S76 (P162)
Burcombe RJ S16 (5.4), S22
(8.1)
Burke F S57 (P107:5)
Burke M S24 (P4)
Burnett AK S55 (P103)
Burrows KE S54 (P99)
Buscombe JR S59 (P113)
Byers R S64 (P130:3)
Caldas C S57 (P107:6)
Callow N S37 (P54)
Cameron D S22 (8.4), S68
(P143), S68 (P144)
Campbell A S28 (P20), S76
(P164), S76 (P165)
Campbell F S32 (P32)
Campbell FC S44 (P72:5), S57
(P107:3), S57 (P107:4), S64
(P130:4), S73 (P157:6), S73
(P157:8), S73 (P157:9), S77
(P165:1)
Campbell LR S23 (8.6), S29
(P23)
Campbell MJ S20 (7.1), S22
(8.3), S25 (P7), S45 (P76:3),
S58 (P108), S66 (P138)
Camplejohn R S16 (5.1)
Cao GW S12 (3.3)
Carlberg C S22 (8.3)
Carmichael J S9 (1.5)
Carpenter R S24 (P3)
Carrington S S61 (P122)
Carroll PS S24 (P2)
Carruthers A S52 (P91)
Cassidy J S12 (3.4), S55 (P102)
Cattell E S28 (P18)
Cawkwell L S28 (P20), S68
(P145), S76 (P164), S76
(P165)
Cemeller S S72 (P157:5)
Chadderton N S49 (P85)
Chakravarti A S20 (7.4)
Chalmers J S44 (P74)
Chau N-M S7 (S22)
Chen J S14 (4.1)
Chester KA S8 (1.2), S63 (P130)
Chesters M S44 (P74)
Chinje EC S18 (6.4), S60 (P119)
Chng HW S16 (5.1)
Chorny VA S49 (P83)
Christodolus K S33 (P35)
Christophorou M S4 (S6)
Chung Y-L S43 (P72:4)
Chung YL S45 (P76:2)
Clamp A S64 (P130:3)
Clamp AR S66 (P137)
Clark AM S18 (6.1), S19 (6.6)
Clark H S34 (P42)
Clark S S45 (P76:2)
Clarke NW S62 (P127)
Clarke P S15 (4.7)
Clarke PA S11 (2.6)
Clarke R S68 (P143), S68 (P144)
Clarke RB S56 (P107:2)
Clawson S S1 (CT2)
Clingen PH S48 (P80:2)
Clive S S40 (P63)
Coad RA S55 (P105)
Coffin R S3 (S4)
Cole C S3 (S2)
Coleman J S39 (P58)
Coleman RE S39 (P58)
Coles C S80 (P176:3)
Coles I S37 (P53)
Collie-Duguid E S14 (4.3), S14
(4.4), S55 (P102)
Collie-Duguid ESR S12 (3.4)
Collins AT S11 (2.5)
Collinson M S30 (P27)
Colston KW S22 (8.3), S25 (P7),
S62 (P125), S63 (P129)
British Journal of Cancer (2004) 91(Suppl 1), S81 - S86
& 2004 Cancer Research UK All rights reserved 0007- 0920/04 $30.00
www.bjcancer.com
Contreras R S63 (P130)
Cook GP S65 (P134)
Coombes RC S44 (P75)
Cooper M S8 (1.4)
Coralli C S60 (P117)
Corbishley C S35 (P45)
Corkhill A S78 (P171)
Cosimo E S19 (6.6), S47 (P78)
Costello E S32 (P32), S61 (P121)
Couchman J S72 (P157:3)
Coulson J S66 (P136)
Coulson JM S14 (4.1)
Cowen R S49 (P85), S50 (P86)
Cowen RL S50 (P88), S50 (P89),
S52 (P92), S60 (P119)
Cox K S78 (P170)
Cranston DW S21 (7.5)
Craven RA S33 (P36)
Crawford S S58 (P109)
Crngorac-Jurcevic T S32 (P32)
Croasdale J S59 (P113)
Croke DT S53 (P96)
Crosby V S41 (P68)
Crowe P S73 (P157:6)
Cullen C S40 (P63)
Cullen MH S1 (CT2)
Cullinane C S17 (5.6)
Cummings J S60 (P116), S75
(P158)
Curry JE S56 (P107)
Curtin N S26 (P11)
Dachs GU S60 (P117)
Dafou D S74 (P157:12), S74
(P157:13)
Dahele M S30 (P25)
Dalal S S17 (5.6), S58 (P110)
Daley F S13 (3.6), S48 (P80:3)
Daley FM S16 (5.4)
Damoto B S54 (P99)
Daniels G S3 (S2)
Danson S S60 (P116)
D'Arcy JA S22 (8.1)
Davies A S75 (P161)
Davies JB S49 (P84)
Davis DJ S56 (P107)
Davis TB S70 (P151)
Dawson M S60 (P116), S75
(P158)
de Wit R S21 (7.7)
Deady R S30 (P24)
Dearnaley D S79 (P176)
Debray C S50 (P86)
Debska G S75 (P160)
Denneny O S60 (P116), S75
(P158)
Dhaliwal H S45 (P76)
Dias-Gunasekara S S44 (P74)
Diaz RM S3 (S2)
Dibling BC S66 (P135)
Dickson J S12 (3.2)
Dive C S4 (S5), S60 (P116), S75
(P158), S75 (P159)
Dixon J S34 (P41)
Dixon N S40 (P63)
Dods JS S33 (P36)
Doherty AP S20 (7.1)
Donnelly ET S73 (P157:6), S77
(P165:1)
Donovan E S80 (P176:3)
Donovan JL S80 (P176:4)
Dorrens J S47 (P77)
dos Santos Pires IM S75 (P159)
Douglas D S20 (7.2)
Douglas DA S36 (P50)
Drew PJ S68 (P145)
D'Souza D S35 (P46)
Du Y S8 (1.1)
Duddalwar V S34 (P40)
Dunn JA S77 (P168)
Durkacz BW S9 (1.6)
Durkin J S75 (P158)
Durocher J S54 (P100)
Durrant LG S22 (8.2), S72
(P157:2)
Dyson P S80 (P176:2)
Earl J S31 (P29), S32 (P31)
Earl R S41 (P65)
Eccles DM S24 (P1)
Eccleston C S14 (4.1)
Eden OB S43 (P72:3)
Eder T S25 (P8), S26 (P9)
Edgson J S67 (P141)
Eisen T S15 (4.8), S21 (7.6), S27
(P15), S27 (P16), S28 (P17),
S8 (1.3)
El-Ghobashy A S39 (P59)
El-Helw L S41 (P67)
El-Khoury B S41 (P68)
Ellershaw C S2 (CT6)
Ellis A S12 (3.1)
El-Mansi M S42 (P69)
El-Tanani M S57 (P107:3), S57
(P107:4), S64 (P130:4), S73
(P157:8), S73 (P157:9)
Emberton M S35 (P44)
Emberton ME S35 (P43)
Erler JT S4 (S5)
Estlin E S48 (P81), S49 (P82),
S68 (P146)
Eustace A S50 (P89)
Evan GI S4 (S6)
Evans A S48 (P81), S49 (P82),
S52 (P91), S68 (P146)
Evans J S31 (P30)
Evans S S35 (P45), S36 (P48),
S36 (P49)
Everard M S43 (P72:1)
Fairbairn LJ S48 (P80:1), S56
(P107:1), S63 (P130:1)
Faisal W S67 (P139)
Faragher AJ S56 (P107:2)
Fearon K S30 (P25)
Fennelly D S70 (P152)
Field JK S12 (3.1), S54 (P99)
Fisher S S44 (P74)
FitzGibbon HJ S74 (P157:10)
Fitzgibbon J S71 (P155)
Flint PJ S52 (P92)
Flower D S69 (P148)
Folkard M S19 (6.7), S47 (P79)
Foran E S53 (P96)
Forbes D S59 (P115)
Ford D S41 (P66)
Forshaw M S3 (S2)
Foster MC S33 (P38)
Fox EJ S70 (P152)
Francis R S8 (1.2), S59 (P113)
Fraser CG S29 (P23)
Freeman A S35 (P44)
Fry AM S56 (P107:2)
Fuerer C S3 (S3)
Fyfe DW S41 (P68)
Gabra H S40 (P63), S54 (P101),
S69 (P147)
Gagen D S63 (P130:1)
Gallagher C S24 (P3)
Gallagher JT S66 (P137)
Gallagher WM S5 (S10), S70
(P152)
Galligan L S9 (1.7)
Ganesan A S63 (P130:2)
Ganguli SJ S62 (P125)
Ganusevich II S49 (P83)
Gao Z S17 (5.5), S52 (P94)
Gao ZQ S12 (3.3), S45 (P76:1)
Garside E S50 (P88)
Garside EJ S49 (P85)
Garvey DJ S47 (P80)
Gayther S S74 (P157:12)
Gayther SA S74 (P157:13)
Gaze M S47 (P78)
Gazzard B S13 (3.7)
Gebril NM S55 (P103)
Geimer M S54 (P100)
Geraghty R S70 (P152)
Germain M S8 (1.2)
Gerty SM S24 (P1)
Gescher AJ S61 (P124)
Ghaneh P S61 (P121)
Gibbens I S42 (P70)
Gilham DG S56 (P107:1)
Gilkes A S55 (P103)
Gillard J S58 (P109)
Gillatt D S35 (P45)
Gillum A S61 (P123)
Gilmore I S31 (P30)
Glennie MJ S64 (P130:5)
Glynne-Jones R S13 (3.6), S34
(P39), S34 (P41), S38 (P57:1)
Gnanapragasam VJ S67 (P142)
Gommersall LM S20 (7.1), S58
(P108)
Goodbrand SA S40 (P61)
Goodlad RA S16 (5.2)
Gore M S21 (7.6), S42 (P70), S8
(1.3)
Gore ME S43 (P72:1)
Gough M S3 (S2)
Gowardhan B S37 (P51)
Gower NH S15 (4.7)
Goyns MH S55 (P104)
Grabowska A S12 (3.2)
Graff C S63 (P130)
Graham J S36 (P49)
Graham JM S36 (P50)
Green A S8 (1.2)
Green AJ S59 (P113)
Green J S53 (P94:1)
Green L S7 (S21)
Greenhalf W S31 (P29), S31
(P30), S32 (P31), S40 (P64)
Grierson I S54 (P99)
Griffin N S8 (1.2)
Griffith JP S49 (P84)
Griffiths JR S43 (P72:4), S45
(P76:2), S46 (P76:4), S46
(P76:5), S46 (P76:6), S46
(P76:7)
Grimer R S41 (P66)
Grimshaw MJ S57 (P107:5)
Guichard S S62 (P126), S76
(P163)
Gumenyuk LD S49 (P83)
Gutierrez MI S55 (P104)
Guy M S22 (8.3), S25 (P7)
Habens F S63 (P130:2)
Hackshaw A S15 (4.7)
Hagan IM S56 (P107:2)
Hagan S S54 (P99)
Hahn S S32 (P31)
Hajek P S43 (P72:2)
Hajiannakis P S30 (P27)
Halkidou K S67 (P142)
Hall E S79 (P176)
Hall J S53 (P97)
Hall JA S23 (8.5)
Hall K S21 (7.6), S43 (P72:1)
Hall S S43 (P72:2)
Hall V S30 (P26)
Hamdi M S33 (P37)
Hamdy FC S80 (P176:4)
Han C S28 (P19)
Hancock B S1 (CT2)
Hancock BW S1 (CT1), S41
(P67)
Hanrahan S S33 (P36)
Harada T S31 (P29)
Harmey J S53 (P94:1)
Harnden P S33 (P36)
Harper PG S15 (4.7)
Harper-Wynne C S15 (4.8)
Harpin RM S78 (P172)
Harris AL S10 (2.2), S10 (2.3),
S17 (5.8), S33 (P35), S43
(P72:4)
Harris JC S11 (2.6)
Harrison M S16 (5.4), S22 (8.1),
S34 (P39), S38 (P57:1), S44
(P73)
Hart C S62 (P127)
Hart I S16 (5.1)
Hart IR S58 (P111)
Hartkamp J S7 (S21)
Hartley JA S48 (P80:2)
Hasan J S64 (P130:3)
Hatton M S77 (P166), S80
(P176:2)
Hatton MQ S39 (P58)
Haviland J S19 (6.8)
Hawkes DJ S15 (4.6)
Hawkins M S27 (P14)
Haycock JW S71 (P157)
Hayne D S33 (P38), S38 (P56)
Hayward DG S56 (P107:2)
Heath JK S72 (P157:4)
Heer R S11 (2.5), S20 (7.2)
Author Index
S82
British Journal of Cancer (2004) 91(Suppl 1), S81 - S86 & 2004 Cancer Research UK
Heighway J S70 (P154)
Held KD S47 (P79)
Hemers E S10 (2.4)
Heng YM S67 (P140), S67
(P141)
Henrioud A S66 (P137)
Herbertson RA S39 (P58)
Hermitage N S68 (P146)
Herrington CS S39 (P59)
Hewer A S73 (P157:6)
Hewison M S25 (P7)
Heywood PL S39 (P59:1)
Hickey DP S33 (P37)
Highley MS S23 (8.6), S29 (P23)
Hill S S30 (P27)
Hill SA S16 (5.3), S60 (P117)
Hilson A S59 (P113)
Hiscott P S54 (P99)
Hitchcock A S44 (P74)
Hockenbery DM S4 (S7)
Hodson A S77 (P166)
Hoh I S35 (P43)
Homewood J S19 (6.8)
Homicsko K S3 (S3)
Honess D S60 (P117)
Honeychurch J S64 (P130:5)
Hong G S54 (P100)
Hoper M S73 (P157:6), S77
(P165:1)
Horsman MR S18 (6.3)
Hoskin P S1 (CT1)
Houghton P S48 (P81), S68
(P146)
Houlston RS S28 (P17)
Howarth A S53 (P95)
Howe FA S46 (P76:4), S46
(P76:5), S46 (P76:6), S46
(P76:7)
Howes N S31 (P30)
Hua ML S76 (P163)
Huang THM S23 (8.5)
Hubner R S42 (P70)
Hughes K S18 (6.1)
Huhalov A S63 (P130)
Hull R S58 (P109)
Hunter AC S75 (P160)
Hussain A S40 (P62)
Hussaini IM S11 (2.7)
Hyland JP S70 (P152)
Iggo R S3 (S3)
Illidge T S8 (1.1)
Illidge TM S64 (P130:5)
Illing RO S21 (7.5)
Illsley M S80 (P176:2)
Imeson J S2 (CT6)
Innes J S39 (P59)
Ireson CR S60 (P117)
Islam S S24 (P4)
Itoh Y S72 (P157:3)
Jackson C S19 (6.8)
Jackson SP S75 (P159)
Jacobs I S74 (P157:12)
Jacobs IJ S74 (P157:13)
Jaffar M S50 (P88), S52 (P93),
S60 (P120)
James LE S15 (4.7)
James M S27 (P16), S8 (1.3)
James ND S20 (7.1)
James P S29 (P23)
James R S13 (3.6)
Janig WG S71 (P156)
Jankowski J S11 (2.6)
Javaherian K S66 (P137)
Jayson GC S64 (P130:3), S66
(P137)
Jenkins JR S70 (P153), S75
(P161)
Jeremiah S S42 (P72)
Jeremiah SJ S39 (P60)
Jiang WG S58 (P108)
Jin D S73 (P157:9)
Jodrell DI S62 (P126), S76
(P163)
Joel SP S59 (P112), S69 (P149),
S70 (P151)
Johnson L S1 (CT3)
Johnson N S26 (P11)
Johnson P S8 (1.1)
Johnson PJ S77 (P168)
Johnson PM S1 (CT2)
Johnston PG S9 (1.7)
Johnston SRD S25 (P5)
Jones C S25 (P6)
Jones DT S10 (2.2)
Jones M S53 (P95)
Jones MA S20 (7.4)
Jones MCM S78 (P172)
Jones R S30 (P24), S36 (P49)
Judson IR S43 (P72:4), S45
(P76:3)
Jurisica I S14 (4.2), S29 (P22:1)
Kadow K S20 (7.4)
Kakkar AK S17 (5.7)
Kalber TL S46 (P76:4), S46
(P76:5)
Kang P S76 (P163)
Kanthou C S60 (P117)
Kassam Z S29 (P21), S34 (P39),
S44 (P73)
Katerinaki E S71 (P157)
Kaur S S72 (P157:5)
Kay EW S33 (P37)
Kaye S S42 (P70)
Kaye SB S43 (P72:1)
Kehoe S S53 (P94:1)
Kelland LR S45 (P76:2)
Kelly J S57 (P107:6)
Kelly S S80 (P176:2)
Kelsey A S49 (P82)
Kelsey AM S43 (P72:3)
Kennedy JE S21 (7.5)
Kenny L S44 (P75)
Kernohan NM S26 (P12)
Kerr K S14 (4.3), S14 (4.4)
Keyzor C S42 (P70), S43 (P72:1)
Khafagy R S62 (P127)
Khanim FL S20 (7.1)
Kharidia R S72 (P157:5)
Khoo V S79 (P176)
Khoudoli GA S73 (P157:7)
Kirk SJ S21 (7.7)
Kitchener H S43 (P72:2)
Knight SC S24 (P4)
Knowles HJ S17 (5.8)
Kong Z S60 (P119)
Koref MFS S70 (P154)
Koruth M S34 (P40)
Kottke T S3 (S2)
Kouokourakis M S10 (2.3)
Kouzarides T S57 (P107:6)
Kovelskaya AV S49 (P83)
Kress R S32 (P31), S40 (P64)
Krippl P S25 (P8), S26 (P10),
S26 (P9), S56 (P106)
Kulbe H S74 (P157:11)
Kumar S S80 (P176:2)
Kumi-Diaka J S58 (P108)
Kushwaha RS S38 (P56)
Kuske B S68 (P143), S68 (P144)
Kyritsi L S53 (P95)
LaCasse E S75 (P158)
Ladroue C S46 (P76:6)
Lalla R S71 (P157)
Lalondrelle S S27 (P14)
Lamb GWA S32 (P33), S32
(P34)
Landau DB S15 (4.5), S15 (4.6)
Lane JA S80 (P176:4)
Lang B S28 (P19)
Langan JE S12 (3.1)
Langdon S S68 (P143), S68
(P144)
Langsenlehner U S25 (P8), S26
(P9), S26 (P10), S56 (P106)
Lankester KJ S16 (5.3)
Lansky EP S58 (P108)
Lawlor B S4 (S6)
Lawrence D S1 (CT3)
Leach MO S16 (5.4), S22 (8.1),
S43 (P72:4), S45 (P76:3)
Leah E S36 (P47)
Leahy DT S70 (P152)
Lee C S21 (7.6)
Lee CP S45 (P76:3)
Leek R S43 (P72:4)
Lees WR S35 (P44)
Lemoine N S31 (P29), S32 (P32)
Lennon F S53 (P96)
Leonard R S22 (8.4)
Leonard RCF S25 (P6), S30
(P24)
Leslie J S31 (P29), S31 (P30),
S32 (P31)
Leung HY S11 (2.5), S20 (7.2),
S36 (P50), S37 (P51), S38
(P55), S67 (P142)
Levack PA S40 (P61)
LeVay J S80 (P176:2)
Levine E S34 (P41)
Lewanski C S29 (P22)
Lewington V S8 (1.1)
Lewis C S53 (P94:1)
Lewis EJ S56 (P107)
Lewis G S60 (P117)
Lewis I S2 (CT6)
Leyton J S45 (P76)
Li N S74 (P157:12)
Li NF S74 (P157:13)
Light AM S33 (P38)
Lilleberg SL S54 (P100)
Linch D S1 (CT1)
Lind MJ S28 (P20), S68 (P145),
S76 (P165)
Lindahl T S71 (P155)
Linehan MW S65 (P131)
Lister A S71 (P155)
Lister TA S59 (P112)
Litago J S45 (P76)
Livingstone A S18 (6.1)
Lloyd S S33 (P36)
Loadman PM S52 (P93), S60
(P120)
Lombard M S31 (P30)
Long AC S53 (P96)
Longley DB S9 (1.7)
Lorigan P S61 (P123)
Lorigan PC S71 (P157)
Lowe LC S25 (P7), S62 (P125)
Lukashev A S3 (S3)
Lunt A S48 (P81)
Lunt SJ S50 (P87)
Lyn E S77 (P166)
Lynagh S S58 (P109)
Macdonald CD S63 (P129)
MacDonald T S7 (S22)
Machin D S2 (CT6)
Mackay H S61 (P122)
MacKean M S23 (8.7), S40 (P63)
Mackenzie I S58 (P111)
Maclennan K S44 (P74)
MacLeod K S68 (P143), S68
(P144)
MacNeil S S71 (P157)
Macpherson JS S62 (P126)
Madhu B S45 (P76:2)
Madhusudan S S33 (P35)
Madjd Z S22 (8.2)
Magor L S56 (P107)
Magrath I S55 (P104)
Maharaj L S59 (P112)
Mairs RJ S18 (6.1), S19 (6.5),
S47 (P77), S47 (P78), S48
(P80:1)
Maisey NR S15 (4.8), S21 (7.6)
Maits RJ S19 (6.6)
Makris A S13 (3.6), S16 (5.4),
S22 (8.1), S34 (P41), S38
(P57:1)
Malats N S31 (P30)
Malinovszky K S25 (P6)
Mandir N S16 (5.2)
Manion M S4 (S7)
Mansi JL S25 (P7), S62 (P125),
S63 (P129)
Mant D S78 (P171)
Marais R S8 (1.3)
Maraveyas A S76 (P164)
Marcora SM S37 (P54)
Margison GP S61 (P123), S70
(P154)
Marshall JF S58 (P111)
Marteau TM S43 (P72:2)
Martin A S34 (P41)
Martin D S26 (P12)
Martin SG S9 (1.5)
Mason C S57 (P107:4)
Mason M S21 (7.7)
Author index
S83
British Journal of Cancer (2004) 91(Suppl 1), S81 - S86
& 2004 Cancer Research UK
Mason MD S1 (CT4)
Matakidou A S28 (P17)
Matakiou A S27 (P15)
Matzow T S52 (P92)
Maughan TS S79 (P173), S79
(P174)
Mawdsley S S13 (3.6)
Maxwell RJ S16 (5.3)
May C S77 (P167)
Mayer A S8 (1.2)
Mayles H S80 (P176:3)
McCann M S64 (P130:4)
McCarthy HO S19 (6.6)
McCluggage WG S73 (P157:6)
McCluskey AG S19 (6.6), S47
(P78)
McCullough S S77 (P165:1)
McDermott U S9 (1.7)
McDowell H S26 (P12)
McElhinney RS S61 (P123)
McFaul C S31 (P29), S31 (P30),
S32 (P31)
McFaul CD S40 (P64)
McGrath H S60 (P116)
McGuigan J S44 (P72:5)
McIntyre DJO S46 (P76:5)
McKay JA S58 (P109)
McKie AB S36 (P50)
McLaren D S37 (P52)
McManus D S75 (P158)
McMillan DC S32 (P33)
McMurry TBH S61 (P123)
McNally RJQ S43 (P72:3)
McNamara A S70 (P153)
McNamara AV S75 (P161)
McNamara JP S45 (P76:3)
McNeill SA S37 (P52)
McPhail J S58 (P109)
McPhail LD S45 (P76:2)
McRonald FE S12 (3.1)
Mead G S8 (1.1)
Mead GM S21 (7.7)
Meadows H S13 (3.6)
Melcher AA S65 (P134)
Mellors C S33 (P38)
Mellows G S8 (1.4)
Menezes C S44 (P72:5)
Merry CLR S66 (P137)
Metheringham R S72 (P157:2)
Michael BD S19 (6.7), S47 (P79)
Middleton MR S21 (7.5)
Mikhaeel G S79 (P175)
Miller EP S54 (P101), S69
(P147)
Miller W S68 (P143), S68 (P144)
Mills I S57 (P107:6)
Mills K S55 (P103)
Milsom MD S48 (P80:1), S63
(P130:1)
Miquel M S15 (4.6)
Mirvish S S44 (P72:5)
Moe M S40 (P62)
Moghimi SM S75 (P160)
Moore CM S35 (P43), S35 (P44)
Moore K S68 (P143), S68 (P144)
Mori N S66 (P136)
Morris RG S42 (P69)
Moss C S70 (P152)
Mosse CA S35 (P43), S35 (P44)
Muir G S35 (P45), S36 (P48)
Mukherjee A S9 (1.5)
Mulcahy HE S70 (P152)
Mulryan K S71 (P157:1)
Murata R S18 (6.3)
Murphy C S57 (P107:3)
Murphy D S4 (S6)
Murray E S22 (8.4)
Murray GI S12 (3.4), S14 (4.3),
S14 (4.4)
Murray JC S51 (P90), S67
(P139), S67 (P140), S67
(P141), S75 (P160)
Murray KE S62 (P128)
Mutch R S28 (P17)
Muthuramalingam SR S33 (P35)
Myatt SS S76 (P162)
Myint S S34 (P41)
Nagase H S72 (P157:3)
Nasir FA S17 (5.7)
Nathan TR S35 (P44)
Naughton C S68 (P143), S68
(P144)
Neal AJ S27 (P14)
Neal D S57 (P107:6)
Neal DE S80 (P176:4)
Neal RD S39 (P59:1)
Nelson M S13 (3.7)
Neoptolemos J S31 (P29), S31
(P30), S32 (P31), S32 (P32)
Neoptolemos JP S40 (P64)
Newbold RF S74 (P157:13)
Newsom-Davis T S13 (3.7)
Nicholson L S16 (5.1)
Nicoll G S26 (P12)
Nicolson M S8 (1.4)
Nicolson MC S14 (4.3), S14 (4.4)
Nilsen H S71 (P155)
Ning HF S12 (3.3)
Nolan IM S70 (P152)
Norbury C S28 (P18)
Norris D S8 (1.4)
Norton A S27 (P16)
Novell R S38 (P57:1)
Noyce J S30 (P26)
Ntougkos E S54 (P101), S69
(P147)
Nunn J S54 (P99)
Nussey F S22 (8.4)
Nystrom M S58 (P111)
O'Brien M S15 (4.8), S27 (P15),
S27 (P16), S27 (P17)
O'Connor M S77 (P165:1)
O'Doherty M S15 (4.5)
O'Donnell A S68 (P143)
O'Donnell J S34 (P42)
O'Donoghue D S70 (P152)
O'Grady A S33 (P37)
Ojha H S33 (P38)
O'Kane SL S28 (P20), S76
(P164), S76 (P165)
Oke AO S69 (P149)
Olijslagers S S36 (P50)
Oliver RTD S21 (7.7)
Oliver SJ S37 (P54)
Oliveros S S30 (P24)
Olsen A S28 (P18)
Omar MM S36 (P50)
O'Neill J S4 (S7)
O'Neill MA S62 (P128)
Opstad KS S46 (P76:6), S46
(P76:7)
O'Reilly M S56 (P107)
Osborne M S73 (P157:6)
Osinsky DS S49 (P83)
Osinsky SP S49 (P83)
Osman S S44 (P75)
Ostler PJ S22 (8.1)
Owen JR S19 (6.8), S80 (P176:2)
Owens J S19 (6.5)
Ozdag H S57 (P107:6)
Packham G S63 (P130:2)
Padhani AR S16 (5.4), S22 (8.1)
Paine M S59 (P114)
Paish EC S22 (8.2)
Panjwani S S67 (P140)
Parker CA S52 (P93)
Parker S S59 (P113), S8 (1.2)
Parsons C S37 (P52)
Parsons KF S65 (P131)
Parton M S15 (4.8)
Patel B S36 (P48)
Patel HK S17 (5.7)
Patel P S33 (P35)
Paul J S23 (8.5)
Payne GS S45 (P76:3)
Payne H S35 (P43), S38 (P56)
Payne HA S35 (P46), S38 (P57)
Pearson ADJ S2 (CT6)
Pearson SJ S70 (P154)
Peckitt C S80 (P176:1)
Pedley RB S16 (5.3), S63 (P130)
Peehl DM S20 (7.1)
Perkins ND S7 (S20)
Perry SE S65 (P134)
Persad R S35 (P45), S36 (P48),
S36 (P49)
Perumal M S45 (P76)
Peto J S80 (P176:1)
Petty RD S14 (4.3), S14 (4.4)
Philips DH S73 (P157:6)
Phillips RM S49 (P84), S52
(P93), S60 (P120), S61
(P122)
Phillips RR S21 (7.5)
Pickering LM S62 (P125), S63
(P129)
Piggott S S80 (P176:1)
Pillai MR S56 (P107:2)
Pinder SE S22 (8.2)
Pinkerton CR S2 (CT6)
Pintilie M S14 (4.2), S29 (P22:1)
Pittam MR S22 (8.1)
Platt-Higgins A S73 (P157:8)
Porter IM S73 (P157:7)
Posner G S64 (P130:4)
Potter GA S61 (P124)
Povey AC S70 (P154)
Powell M S79 (P176)
Powell NG S39 (P60), S42 (P72)
Power D S37 (P53)
Power DA S29 (P22)
Powles T S13 (3.7)
Preston T S30 (P25)
Priest K S27 (P16)
Prime W S31 (P29), S32 (P32),
S39 (P59)
Primrose JN S78 (P171)
Prise K S18 (6.1)
Prise KM S19 (6.7), S47 (P79),
S48 (P80:2)
Pritchard DM S10 (2.4)
Protheroe A S33 (P35)
Protheroe AS S21 (7.5)
Prouse A S42 (P70)
Puntis MCA S71 (P156)
Purcell W S62 (P128)
Pyle L S8 (1.3)
Pyrah ITG S16 (5.2)
Qian W S1 (CT1)
Qiao J S3 (S2)
Quirke P S65 (P134)
Rabbitts P S6 (S17)
Rabiasz GJ S54 (P101), S69
(P147)
Radford J S1 (CT2)
Radford JA S1 (CT1)
Ramage JM S72 (P157:2)
Ramani V S62 (P127)
Rankin EM S8 (1.4), S23 (8.6),
S29 (P23), S41 (P65), S59
(P114), S59 (P115), S64
(P130:6)
Ranson M S60 (P116), S61
(P123), S75 (P158)
Ratcliffe P S10 (2.2)
Ratcliffe PJ S17 (5.8)
Ravichandran D S22 (8.1)
Rawlings C S80 (P176:3)
Raynaud FI S45 (P76:3)
Rayter S S5 (S11)
Real P S31 (P30)
Reddy J S4 (S6)
Regan J S19 (6.8)
Richman PI S22 (8.1)
Rickards D S35 (P43)
Rieder H S32 (P31), S40 (P64)
Rigg A S42 (P70)
Risk JM S12 (3.1), S54 (P99)
Rmali K S71 (P156)
Roberts S S7 (S21)
Robertson N S10 (2.3)
Robinson SP S45 (P76:2), S46
(P76:4), S46 (P76:5)
Robson CN S11 (2.5), S20 (7.2),
S36 (P50), S37 (P51), S38
(P55), S67 (P142)
Robson T S19 (6.6)
Rogers P S7 (S22)
Ronan J S22 (8.2)
Ross S S18 (6.1), S47 (P77)
Ross SC S19 (6.5)
Rothkamm KM S48 (P80:3)
Rowbottom L S12 (3.1)
Rowland JG S38 (P55)
Rudd RM S15 (4.7)
Ruddle RR S45 (P76:3)
Rudland PS S73 (P157:8)
Author Index
S84
British Journal of Cancer (2004) 91(Suppl 1), S81 - S86 & 2004 Cancer Research UK
Rustin GJS S16 (5.3), S21 (7.7)
Rutten F S44 (P74)
Ryan AJ S16 (5.2), S46 (P76:4),
S46 (P76:5)
Ryan C S27 (P16)
Ryan D S24 (P3)
Ryan R S53 (P94:1)
Ryder D S1 (CT2)
Rye T S40 (P63)
Saber M S51 (P90)
Saha V S70 (P151)
Saini A S27 (P16)
Sale S S61 (P124)
Samonigg H S25 (P8), S26 (P9),
S26 (P10), S56 (P106)
Samson LD S3 (S1)
Samuel L S34 (P41)
Samuel LM S12 (3.4), S34 (P40)
Sanchez-Perez L S3 (S2)
Sanders C S54 (P100)
Sanjoy P S27 (P15)
Santos K S49 (P82)
Saraga G S61 (P121)
Sargent JM S70 (P151)
Saunders MP S52 (P92)
Saxena A S72 (P157:5)
Saxena S S72 (P157:5)
Saxton W S37 (P54)
Scholes AGM S54 (P99)
Schwartz B S8 (1.3)
Schwartz P S4 (S7)
Scott D S54 (P101)
Seabra l S53 (P95)
Sebag-Montefiore D S34 (P41)
Seiki M S72 (P157:3)
Selby PJ S33 (P36)
Sellar GC S54 (P101), S69
(P147)
Severn O S35 (P46), S38 (P57)
Shaaban A S39 (P59)
Shah N S29 (P21)
Shah RNH S43 (P72:1)
Shankland SH S79 (P173)
Shao C S19 (6.7), S47 (P79)
Sharma SK S8 (1.2)
Shaw JM S12 (3.1)
Sheahan K S70 (P152)
Sheard V S48 (P80:1)
Shekouh A S32 (P32)
Shepherd FA S29 (P22:1)
Shepherd NA S55 (P105)
Sheppard F S52 (P91)
Sheppard FC S50 (P88)
Shi YH S45 (P76:1)
Shima D S60 (P117)
Shnyder SD S64 (P130:3)
Short SM S38 (P57:1)
Shousha S S44 (P75)
Sibson R S31 (P29)
Siemann DW S5 (S12)
Simmonds PD S24 (P1)
Simon JW S38 (P55)
Simth IE S27 (P15)
Sina-Frey M S32 (P31), S40
(P64)
Singh M S72 (P157:5)
Singh S S18 (6.4), S60 (P119)
Slabas AR S38 (P55)
Slade M S28 (P18)
Sloan J S44 (P72:5)
Smart H S31 (P30)
Smith AG S33 (P37)
Smith I S27 (P16)
Smith IE S25 (P5)
Smith L S76 (P164), S76 (P165)
Smith P S1 (CT1), S1 (CT2)
Smyth J S68 (P144)
Smyth JF S23 (8.7), S40 (P63),
S54 (P101), S69 (P147)
Solano J S35 (P46)
Somani BK S20 (7.4), S33 (P38),
S38 (P56)
Sothi S S41 (P66)
Soucek L S4 (S6)
Souhami RS S15 (4.7)
Southgate T S48 (P80:1)
Spendlove I S22 (8.2), S72
(P157:2), S74 (P157:10)
Spicer J S5 (S11)
Spiro SG S15 (4.7)
Spooner D S41 (P66)
Squires MS S56 (P107)
Staffurth JN S79 (P176)
Stanley AJ S33 (P36)
Stater D S41 (P67)
Stead M S78 (P169)
Stec J S59 (P112)
Steele A S60 (P117)
Steiner J S8 (1.4)
Stenning SP S1 (CT2), S21 (7.7)
Stephens LA S39 (P60), S42
(P72)
Stephens R S77 (P166)
Stephens TC S62 (P127)
Stern PL S71 (P157:1)
Stevens MFG S9 (1.5)
Stevens T S78 (P169)
Stevenson HP S29 (P23)
Stevenson M S73 (P157:6)
Steward WP S61 (P124)
Stewart DS S12 (3.4)
Stewart M S23 (8.7)
Stewart S S37 (P53)
Stewart VC S19 (6.7)
Stirling JJ S16 (5.4), S22 (8.1)
Stone M S58 (P111)
Storey DJ S40 (P63)
Stratford I S48 (P81), S60
(P120), S68 (P146)
Stratford IJ S4 (S5), S18 (6.4),
S47 (P80), S49 (P85), S50
(P86), S50 (P87), S50 (P88),
S50 (P89), S52 (P91), S52
(P92), S52 (P93), S60 (P119)
Strathdee G S53 (P97)
Strauss SJ S59 (P112)
Stronach EA S54 (P101)
Stuart NSA S37 (P54)
Stubbs M S43 (P72:4)
Subramaniam R S38 (P57)
Sue A S27 (P15)
Sugarbaker DJ S6 (S18)
Sully C S8 (1.2)
Sumpter K S15 (4.8)
Sunderland G S66 (P136)
Sun-Mynt H S1 (CT1)
Sun YQ S12 (3.3)
Swann PR S47 (P80)
Swarbrick C S44 (P72:5)
Swedlow JR S73 (P157:7)
Swigart LB S4 (S6)
Sydenham M S79 (P176), S80
(P176:2), S80 (P176:3)
Sydenham MA S80 (P176:1)
Sydes MR S1 (CT2)
Symonds I S44 (P74)
Symonds P S51 (P90), S67
(P139), S67 (P141), S75
(P160)
Syndikus I S80 (P176:2)
Szewczyk A S75 (P160)
Szlosarek PW S57 (P107:5)
Taher T S71 (P157:1)
Tait D S79 (P176)
Talbot D S28 (P18), S28 (P19)
Tan KW S47 (P77)
Tan SK S45 (P76:3)
Taylor KJ S54 (P101)
Taylor NJ S16 (5.4), S22 (8.1)
Taylor S S10 (2.1)
Telfer BA S47 (P80), S50 (P88),
S50 (P89)
Teodoridis JM S53 (P97)
terHaar GR S21 (7.5)
Terschendorff A S57 (P107:6)
Thirlwell C S13 (3.7)
Thomas C S29 (P21), S34 (P39),
S44 (P73), S53 (P95)
Thomas DR S33 (P38)
Thomas G S30 (P27)
Thomas GA S18 (6.2), S25 (P6),
S39 (P60), S42 (P72), S73
(P157:6)
Thomas GJ S58 (P111)
Thomas H S8 (1.4)
Thomas Y S27 (P15)
Thompson AM S26 (P12), S40
(P61), S62 (P128)
Thompson C S32 (P32)
Thompson J S3 (S2)
Thomson G S26 (P12)
Thomson GA S62 (P128)
Thomson J S40 (P61)
Threadgold J S31 (P30)
Timotheadou E S21 (7.6)
Timson G S55 (P104)
Tobin MJ S44 (P74)
Tolner B S8 (1.2), S63 (P130)
Tonks A S55 (P103)
Totty N S33 (P36)
Townsend K S25 (P7)
Toy L S30 (P27)
Tozer G S60 (P117)
Tozer GM S16 (5.3)
Tracy M S45 (P76:3)
Trask C S15 (4.7)
Treasure T S15 (4.5), S80
(P176:1)
Tronko MD S39 (P60), S42
(P72)
Trott DA S74 (P157:13)
Troy H S43 (P72:4)
Tsao MS S14 (4.2), S29 (P22:1)
Tselepis C S66 (P138)
Tunstall RG S61 (P124)
Turley H S10 (2.2)
Turner AJ S36 (P47)
Turner KJ S37 (P52)
Turner LE S27 (P13), S78 (P170)
Tutt A S8 (1.1)
Tuttle RM S42 (P72)
Uekita T S72 (P157:3)
Underhill-Day N S72 (P157:4)
Unwin RD S33 (P36)
Usmani BA S36 (P47)
Vaisanen S S22 (8.3)
Valkovskaya NV S49 (P83)
van As N S79 (P175)
van Delft F S70 (P151)
Varro A S10 (2.4)
Vasey PA S32 (P33), S32 (P34),
S33 (P35), S53 (P97)
Veerakumarasivam A S57
(P107:6)
Venables K S80 (P176:3)
Verborg WA S23 (8.6)
Vervecken W S63 (P130)
Veuger SJ S9 (1.6)
Vigushin DM S44 (P75)
Vile RG S3 (S2)
Vimalachandran D S32 (P32)
Vincent A S28 (P19)
Vinnicombe S S24 (P3)
Violet JA S59 (P113)
Volpato M S60 (P120)
Von der Maase H S21 (7.7)
Wagner K S7 (S21)
Walewski J S1 (CT2)
Walker G S41 (P68)
Wall L S30 (P25)
Waller D S80 (P176:1)
Walters KB S54 (P100)
Wang B S12 (3.3), S17 (5.5), S45
(P76:1), S52 (P94)
Wang W S55 (P102)
Warburton HE S65 (P131)
Ward CJ S59 (P114), S59 (P115),
S64 (P130:6)
Ward TH S60 (P116), S75
(P158)
Warenius H S53 (P95)
Warnberg F S9 (1.8)
Warner PJ S55 (P105)
Waterfall J S8 (1.4)
Waterston A S13 (3.7)
Waterton JC S46 (P76:4), S46
(P76:5)
Watkins D S27 (P15), S27 (P16)
Watkins JL S34 (P42)
Watson AJM S70 (P153), S75
(P161)
Watson MB S68 (P145)
Watson R S32 (P32)
Watson SA S11 (2.6), S12 (3.2)
Webster S S69 (P150)
Wedge SR S16 (5.2)
Wei SH S23 (8.5)
Author index
S85
British Journal of Cancer (2004) 91(Suppl 1), S81 - S86
& 2004 Cancer Research UK
Wells P S79 (P176)
Westwell AD S9 (1.5)
Whipp L S80 (P176:2)
White D S9 (1.8)
White R S53 (P95)
White RJ S6 (S19)
Whitney LK S50 (P87)
Whittaker L S46 (P76:5)
Wigle DA S14 (4.2), S29 (P22:1)
Wilbanks G S74 (P157:12)
Wilbanks GD S57 (P107:5)
Wilkie E S41 (P65)
Wilkinson RW S16 (5.2)
Williams ARW S42 (P69)
Williams DW S42 (P72)
Williams ED S73 (P157:6)
Williams K S49 (P85)
Williams KJ S5 (S13), S50 (P86),
S50 (P87), S50 (P88), S50
(P89), S52 (P91), S52 (P92),
S52 (P93), S60 (P119)
Williams MV S1 (CT1)
Williamson AJK S66 (P135)
Williamson CJ S70 (P151)
Wilson D S41 (P66)
Wilson G S13 (3.6)
Wilson JL S57 (P107:5), S74
(P157:11)
Wilson L S18 (6.1)
Wilson R S78 (P169)
Wittrup KD S63 (P130)
Wolf CR S59 (P114), S59 (P115),
S64 (P130:6)
Woll PJ S69 (P148), S69 (P150)
Wolstenholme V S79 (P175)
Wong T S31 (P30)
Wong W S44 (P73)
Wood EM S39 (P59:1)
Wood K S37 (P53)
Woodhead A S56 (P107)
Woodman AC S34 (P42), S54
(P98), S55 (P105)
Woolford LB S56 (P107:1), S63
(P130:1)
Workman P S7 (S22), S45
(P76:3)
Wrightham E S38 (P56)
Wu F S21 (7.5)
Wu J S48 (P80:2)
Wyatt PG S56 (P107)
Wynn R S49 (P82)
Xu H S12 (3.3)
Yaghjyan L S42 (P71)
Yan L S31 (P30), S32 (P31)
Yarnold J S1 (CT3), S19 (6.8),
S80 (P176:2), S80 (P176:3)
Yeomans NP S49 (P84)
Young LS S66 (P138)
Yu DX S12 (3.3), S17 (5.5), S45
(P76:1), S52 (P94)
Yu J S59 (P114), S64 (P130:6)
Yurchenko OV S49 (P83)
Yurek-George A S63 (P130:2)
Zalutsky MR S19 (6.6), S47
(P77)
Zhi C S54 (P100)
Zhu YL S17 (5.5)
Zhu YM S69 (P148), S69
(P150)
Zied NA S60 (P120)
Zivanovic M S8 (1.1)
Author Index
S86
British Journal of Cancer (2004) 91(Suppl 1), S81 - S86 & 2004 Cancer Research UK

				

